# 

**Published:** 1895-11

**Authors:** 


### BOOKS RECEIVED.

Medical Diagnosis with Special Reference to Practical Medicine. A Guide to the Knowledge and Discrimination of Diseases. By J. M. DaCosta, M. D., LL. D., President of the College of Physicians of Philadelphia; Emeritus Professor of Practice of Medicine and of Clinical Medicine at the Jefferson Medical College, Philadelphia; Physician to the Pennsylvania Hospital, etc. Octavo, pp. 1104. Illustrated with engravings on wood. Eighth edition, revised. Philadelphia: J. B. Lippincott Company. 1895.

Cutaneous Medicine. A Systematic Treatise on the Diseases of the Skin. By Louis A. Duhring, M. D., Professor of Diseases of the Skin in the University of Pennsylvania, etc. Octavo, pp. vii.—221. Illustrated. Philadelphia: J. B. Lippincott Company. 1895.

Transactions of the Association of American Physicians. Tenth session, held at Washington, D. C., May 30 and 31, 1895. Philadelphia: Wm. J. Dornan, Printer. 1895.

The Pathology and Surgical Treatment of Tumors. By N. Senn, M. D., Ph. D., LL. D., Professor of Practice of Surgery and Clinical Surgery, Rush Medical College; Professor of Surgery, Chicago Polyclinic; Attending Surgeon to Presbyterian Hospital; Surgeon-in-Chief, St. Joseph's Hospital, Chicago. Octavo, pp. 709. Illustrated by 515 engravings, including full-page colored plates. Price, cloth, $6.00; half-morocco, $7.00. Philadelphia: W. B. Saunders, 925 Walnut street. 1895.

A Treatise on Nervous and Mental Diseases. By Landon Carter Gray, M. D., Professor of Diseases of the Mind and Nervous System in the New York Polyclinic. New (2d) edition, In one very handsome octavo volume of 728 pages, with 172 engravings and three colored plates. Cloth, $4.75; leather, $5.75. Philadelphia: Lea Brothers & Co., Publishers. 1895.

A Hand-Book of Medical Diagnosis. By James B. Herrick, M. D., Adjunct Professor of Medicine, Rush Medical College, Chicago. In one handsome 12mo volume of 429 pages. with eighty engravings and two colored plates. Cloth, $2.50. Philadelphia: Lea Brothers & Co., Publishers. 1895.

The Pathology and Treatment of Venereal Diseases. By Robert W. Taylor, A. M., M. D., Clinical Professor of Venereal Diseases in the College of Physicians and Surgeons, New York. In one very handsome octavo volume of 1002 pages, with 230 engravings and seven colored plates. Cloth, $5.50; leather, $6.50. Philadelphia: Lea Brothers & Co., Publishers. 1895.

A Text-Book of Practical Medicine. Designed for the use of Students and Practitioners of Medicine. By Alfred L. Loomis, M. D., LL. D., Professor of Pathology and Practical Medicine in the Medical

Department of the University of the City of New York; Visiting Physician to Bellevue Hospital, etc. Revised and enlarged, with 207 illustrations and one chromo-lithographic plate. Eleventh edition. 1134 pages. Price, cloth, $6.00; leather, $7. New York: William Wood & Co. 1895.

Text-Book of Physiology. By Michael Foster, M. D., F. R. S., Prelector in Physiology and Fellow of Trinity College, Cambridge, Eng. New (sixth) American edition with notes and additions. In one handsome octavo volume of 922 pages, with 257 illustrations. Cloth, $4.50; leather, $5.50. Philadelphia: Lea Brothers & Co., Publishers. 1895.

Modern Materia Medica with Therapeutic Notes. For the use of Practitioners and Students of Medicine. By Dr. Otto Roth. Seventh edition. Revised by Dr. Gregor Smith, Würzburg. One volume of 467 pages, octavo, muslin binding. Price, $2.00. New York: William Wood & Co. 1895.

